# Changes in the effects of heat on mortality among the elderly from 1998–2010: results from a multicenter time series study in Italy

**DOI:** 10.1186/1476-069X-11-58

**Published:** 2012-09-03

**Authors:** Patrizia Schifano, Michela Leone, Manuela De Sario, Francesca de’Donato, Anna Maria Bargagli, Daniela D’Ippoliti, Claudia Marino, Paola Michelozzi

**Affiliations:** 1 Department of Epidemiology, Lazio Regional Health Service, Via di Santa Costanza, Roma, 53, 00198, Italy

**Keywords:** Prevention plan, Heat, Non linear model, Mortality, Elderly

## Abstract

**Background:**

This multicenter study is aimed at estimating changes in the effect of high temperatures on elderly mortality before and after the 2003 heat waves and following the introduction of heat prevention activities.

**Methods:**

A total of sixteen cities were included in the study. City-specific relationships between maximum apparent temperature (MAT) and elderly daily mortality before (1998–2002) and after (2006–2010) intervention were modelled through non-linear distributed lag models and estimates were combined using a random effect meta-analysis. We estimated the percentage change in daily mortality for 3°C variations in MAT above the 25^th^ percentile of the June city-specific 1998–2002 distribution.

A time-varying analysis was carried out to describe intra-seasonal variations in the two periods.

**Results:**

We observed a reduction in high temperatures’ effect post intervention; the greatest reduction was for increases in temperature from 9°C to 12°C above the 25^th^ percentile, with a decrease from +36.7% to +13.3%. A weak effect was observed for temperatures up to 3°C above the 25^th^ percentile only after. Changes were month-specific with a reduction in August and an increase in May, June and September in 2006–2010.

**Conclusions:**

A change in the temperature-mortality relationship was observed, attributable to variations in temperature distributions during summer and to the introduction of adaptation measures. The reduction in the effect of high temperature suggests that prevention programs can mitigate the impact. An effect of lower temperature remains, indicating a relevant impact of temperature at the beginning of summer when the population has not yet adapted and intervention activities are not fully operational.

## Background

Reducing the effects of heat on health is one of the priorities identified by the WHO [1]. Changes in the effects of heat on human health depend not only on changes in levels of heat exposure, but also on variations in the characteristics of populations which confer a greater susceptibility and in the adaptive capacity due to individual and community efforts [2]. Studies mainly from the US have documented a decline in heat-related mortality in the last decades [3-6], which has been attributed to technological improvements and to changes in socio-demographic characteristics of the population, or to adaptive measures. In Europe evidence of time variations in heat-related mortality is limited and the few studies have mainly focused on comparing the impact of the 2003 heat waves with that of heat waves in the following years [7-10]. In several European countries after 2003, warning systems and a number of specific public health interventions have been widely introduced, however the actual level of implementation of different response measures was heterogeneous [11,12], and evidence of the effectiveness of specific preventive activities is still lacking [11,13,14].

Evaluating the effectiveness of heat–health action plans is extremely difficult for several reasons. First of all, programs within the same country are very heterogeneous and the same program can vary from year to year. Furthermore, it is difficult to plan a study design able to take into account all the potential confounders and effect modifiers of a plan’s effectiveness. Randomized trials (RCT) are very difficult in this context for ethical and organizational reasons [15], and, observational studies have important limits due to the identification of a proper control population and to the type of intervention to be evaluated. However, it is necessary from both an epidemiological and a public health point of view to give a description of changes occurring in the heat-mortality relationship over time to allow public health decision makers to make evidence-based choices, to better allocate available resources and to address future research to the most urgent problems.

In Italy, a national program for the prevention of the effects of heat on subjects aged 65 years and older has been operational since 2004, supported by the Department of Civil Protection and the Ministry of Health [12]. To date, it includes 34 major cities, which differ in climate and socio-demographic characteristics, and covers 21% of Italian residents aged 65 years and over.

The main components of the program are city-specific heat health watch warning systems (HHWWS), national prevention guidelines on which local prevention measures are developed, the identification of susceptible subgroups on whom prevention activities are targeted and the definition of a rapid mortality surveillance system. Although there are differences between prevention plans at the local level the key component is the active surveillance of high risk patients by general pratictioners (GPs). When level 2 and level 3 warnings are issued by the HHWWS, GPs monitor their patients through telephone calls and home visits, and provide specific interventions such as modulation of pharmacological treatment, requesting for home-based treatment and giving special attention to high risk patients recently discharged from hospitals. Details on the Italian heat plan are reported elsewhere [12].

The present multicenter study evaluates temporal changes in the impact of summer temperatures on mortality among those older than 65 years, before and after the introduction of the national heat health prevention program in Italy.

## Materials and Methods

### Data

The study includes data on residents of 16 of the 34 Italian cities included in the National Prevention Program. We selected cities that have had an active HHWWS and local prevention plan since summer 2004 and have complete temperature and mortality time series for the whole study period. The study was restricted to subjects over 65 years of age as prevention programs are targeted to the elderly.

### Study period

The study period includes 1998 to 2002, as the period preceding the introduction of the heat prevention program (pre-intervention), and from 2006 to 2010 as the period following their implementation (post-intervention). We excluded 2003 because of the exceptional meteorological conditions and the related mortality burden observed in many Italian cities [16,17], and years 2004 and 2005 because prevention programs were not fully operational. All analyses were restricted to the warm season defined as the period between May 15th and September 15th.

### Outcome measure

The study outcome is residents’ daily mortality from any natural cause that occurred in the selected cities (International Classification of Disease, ICD-9 1–799); data were extracted from local mortality registries and the rapid mortality surveillance system [12].

#### Exposure measure

Exposure was measured by daily maximum apparent temperature (MAT) defined as the maximum value between 1200UTC and 1800UTC. Apparent temperature is a discomfort index based on air and dew point temperature [18,19].

Meteorological data refer to the airport station located closest to the city centre and were obtained from the Meteorological Service of the Italian Air Force.

#### Statistical Analysis

The city-specific MAT mortality relationship was explored using a Generalized Additive Model (GAM) model and the overall relationship was discerned through a multivariate meta-analysis.

The analysis was developed in two steps. First a common model was applied to each city to obtain city-specific estimates, and then estimates were combined using a random effect meta-analysis.

It is well known that the strength of the association between temperature and mortality varies according to temperature levels, assuming approximately a J-shaped relationship along the temperature axis. We adopted a non-linear approach to accurately model the temperature-mortality association along the whole range of summer temperatures, providing specific estimates for each temperature value. We used a non-linear distributed lag model (DLNM) with a Poisson link function, to overcome linearity and simultaneously taking into account the delayed effect of exposure [20].

The mortality-temperature relationship was modelled using a cubic natural spline with six degrees of freedom (df), while the temporal structure was modelled constraining the curve to follow a cubic natural spline with five degrees of freedom. A lag window of up to seven days was selected because it has been suggested that it accurately describes the delayed effect of exposure [21]. A preliminary analysis on data from 1998–2002 was carried out using a constrained lag model to define the significant lag structure in each city.

To compare city-specific effects, an exposure indicator was defined based on the difference between daily MAT and a city-specific reference point, corresponding to thermal comfort. The city-specific reference point was defined as the 25th percentile of the MAT summer distribution in the period 1998–2002. We give results for three-degree step increases in MAT above the reference point.

We adjusted for seasonality and long time-trends using indicator variables for days of the week, holiday and month and linear quadratic terms of time, respectively.

In the second step, for each of the temperature intervals previously described, a pooled estimate from all cities was computed with a random effect meta-analysis based on restricted maximum likelihood [22].

Furthermore, in order to understand changes in seasonality of extreme temperature over the study period, a multivariate meta-analytic time-varying analysis was carried out [23]. We estimated the effect of 1°C increases in MAT over the 25^th^ city-specific percentile considering a common 0–3 lag for all cities. We assumed that the effect of the exposure follows a sine and cosine function, according to Baccini et al. [24].

A sensitivity analysis was carried out excluding some cities from the meta-analysis. In particular, small cities that might produce an unreliable overall estimate were excluded, as were those with a temperature/mortality trend that differed greatly from the others, because they might increase heterogeneity.

The study did not include human subjects.

## Results

### Data Set

We included 16 cities in the study that fulfilled the required criteria (Table 1). The number of inhabitants ranges from about 50,000 in Campobasso and Civitavecchia to over 2,5 million in Rome; the percentage of the population over 65 years varies roughly between 14% in Latina and Palermo to 26% in Trieste, Bologna, Genova and Trieste. The daily mean number of deaths in 65+ year olds in the analysed period ranges from less than 1 in Campobasso and Civitavecchia to 45 in Rome (Table 1).

**Table 1:** Population size, daily 65+ mortality, maximum apparent temperature (MAT) and lag by city

**City**	All ages population*	Percentage (%) of 65+ yr olds*	**Daily deaths, 65+ yr olds°**		**25th pct of MAT (°C)**		**LAG§**
			**Mean**	**SD**	**1998-2002**	**2006-2010**	
Trieste	210882	26.0	6.7	2.7	23.8	24.3	6
Brescia	187188	21.4	4.2	2.2	23.8	24.6	3
Milan	1253503	22.9	21.6	6.1	26.2	24.4	3
Verona	253267	21.2	5.2	2.5	24.9	24.9	4
Venezia	270963	23.8	6.3	2.6	23.8	24.1	4
Turin	864671	22.5	15.5	5.4	22.2	22.4	4
Bologna	370363	26.6	9.1	3.3	25.1	24.7	3
Genova	609399	25.7	17.0	4.4	25.6	24.7	3
Florence	355315	25.7	8.8	3.2	26.1	25.2	3
Viterbo	59263	19.9	1.1	1.0	24.4	24.5	4
Civitavecchia	49966	17.4	0.7	0.9	26.4	28.6	2
Rome	2545860	19.0	45.1	9.5	26.0	26.4	3
Campobasso	50826	17.6	0.7	0.9	19.6	20.0	2
Latina	108195	14.1	1.4	1.2	26.9	27.2	1
Bari	316278	17.2	5.3	2.4	25.6	25.3	3
Palermo	686045	14.7	11.1	3.9	26.6	26.8	3

* 2002 resident population (National Institute of Statistics. http://www.demo.istat.it).

§ City-specific lags from the distributed lag model; 1998–2002. ° Over 1998–2002 period.

### Temperatures Variations

**Figure 1:**
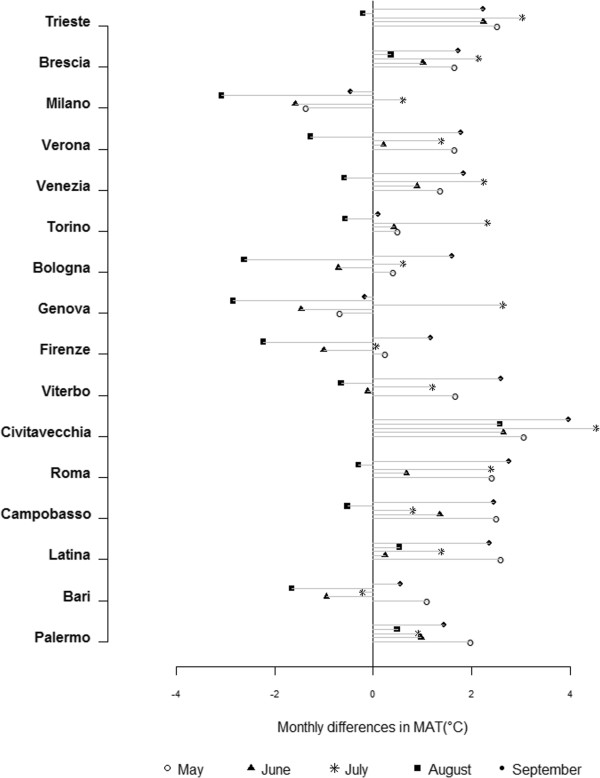
City-specific monthly difference in 75th percentile values of MAT pre (1998–2002) and post (2006–2010) intervention.

The 25th percentile value of the MAT distribution pre intervention varied between 24°C and 27°C with the exception of the city of Campobasso, with a value of 20°C, and Turin, with a value of 22°C. The city-specific lag value ranged from 1 to 6 days (Table 1). No important differences in the two periods have been observed. Monthly differences in the 75^th^ percentile of city-specific MAT distribution in the two periods are reported in Figure 1.

The figure shows a shift towards higher values in most cities, with a greater increase during the months of May (with the only exception of the city of Milan and Genoa), July (with the exception of Bari) and September (with the exception of Milan and Genova). MAT percentiles values for the month of June are quite similar for the two periods, while in August values are lower post intervention in all cities except for Brescia, Civitavecchia, Latina and Palermo.

### City-specific estimates

**Figure 2:**
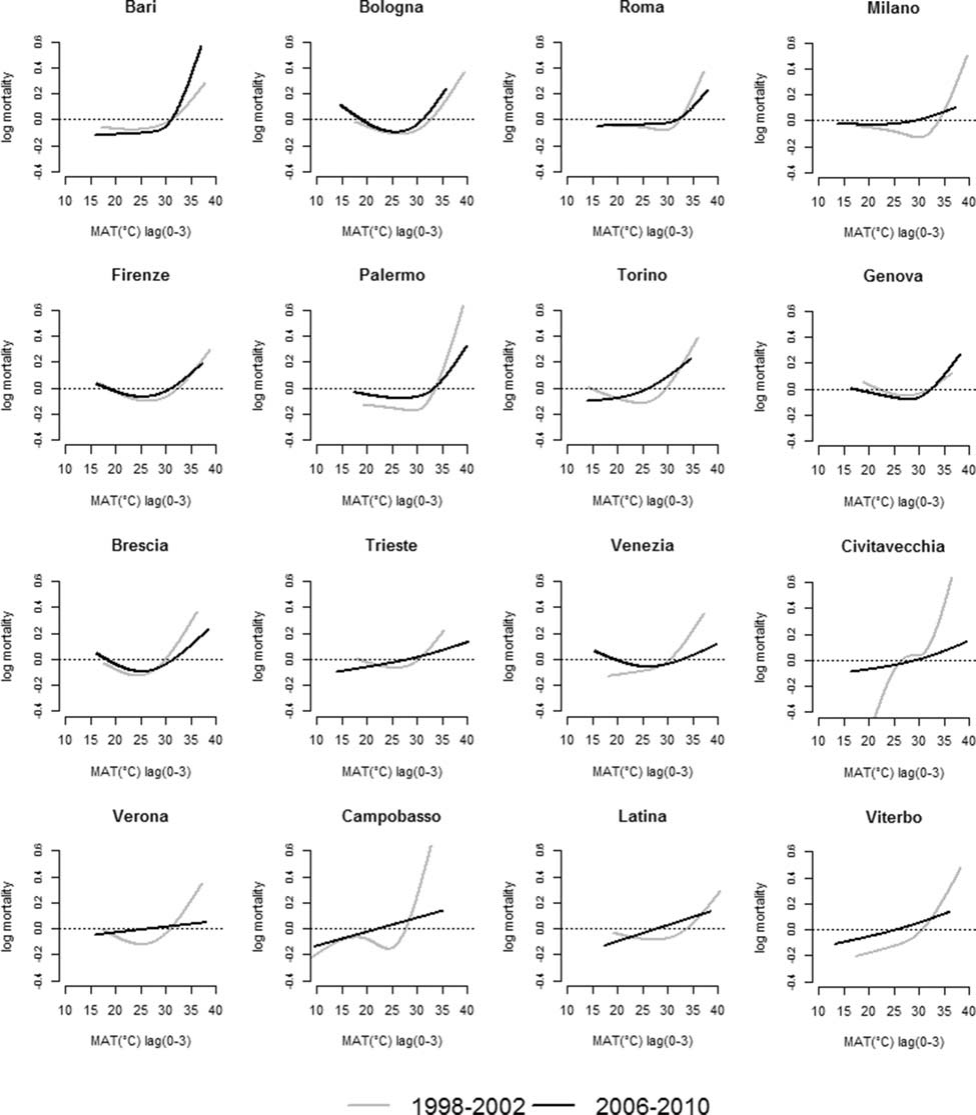
City-specific MAT (°C)- daily 65+ mortality relationships pre (1998–2002) and post (2006–2010) intervention.

We observed a weaker relationship between heat and mortality in almost all cities post-intervention than in the pre-intervention period (Figure 2), furthermore, a decrease in extremely high temperature values post intervention was observed. Only in Bari and, to a lesser extent in Genoa, did the effect of high temperatures seem to be stronger. No differences could be detected in Florence and Bologna. In some cases the temperature-mortality relationship became almost linear (Trieste, Civitavecchia, Verona, Campobasso, Latina and Viterbo).

During the post-intervention period the lag period shortened in most cities (results not shown).

### Overall results

**Figure 3:**
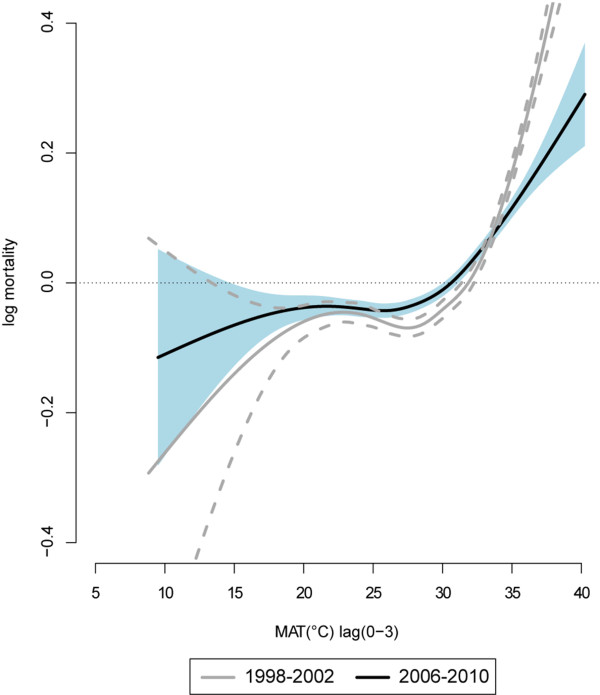
Overall MAT (°C)-daily 65+ mortality relationship pre (1998–2002) and post (2006–2010) intervention.

In Figure 3 the meta-analytic curves of the two periods are reported. The before-after trend more commonly detected in the city-specific curves is shown: a very slight increase in the effect of lower temperatures and a clear reduction of the effect of high temperatures on mortality in the post-intervention period. Also it shows how the upper limit of observed temperatures was lower in the 2006–2010 period.

Dividing the temperature range into three-degree intervals, for each study period we obtained five temperature intervals and five estimates of the exposure–mortality relationship in terms of percent change in mortality risk. City-specific estimates are reported in the Web appendix (Additional file 1:). The test of heterogeneity resulted significant for three of them, specifically between 6°C and 12°C over the reference pre intervention and between 3°C and 6°C over the reference post intervention (data not shown).

Table 2 reports the overall estimates of the exposure–mortality relationship in terms of percent change in mortality risk per 3°C of temperature increase starting from the city- specific reference value. A non-significant increase in mortality was observed up to six degrees above the reference value before the intervention and a weak increase post intervention. Six degrees above the reference value corresponds approximately to the 75th percentile of the city-specific MAT distribution. For temperature increases above this point, a significant reduction in the effect of heat was detected after the intervention: the highest reduction was observed between 9°C and 12°C above the reference value where the percent change dropped from 37% to 13%.

**Table 2:** Overall effect of maximum apparent temperature (MAT) on daily 65+ mortality pre (1998–2002) and post (2006–2010) intervention

	**1998-2002**		**2006-2010**	
MAT Increase*	**% change**^**^**^	**95% CI**	**% change**^**^**^	**95% CI**
0 to 3	5.65	−3.82-16.07	5.65	0.60 - 10.96
3 to 6	6.72	−2.57 - 16.77	7.79	−0.60 - 16.88
6 to 9	24.73	14.22 - 36.21	15.60	10.08 - 21.41
9 to 12	36.75	25.73 - 48.88	13.31	6.50 - 20.56
12 to 15	41.76	21.41 - 65.53	5.65	−5.54 - 18.06

* Percent change for 3°C increases in MAT over the 25th city-specific percentile.

^^^ Random effect meta-analysis pooled estimates.

### Time varying analysis

**Figure 4:**
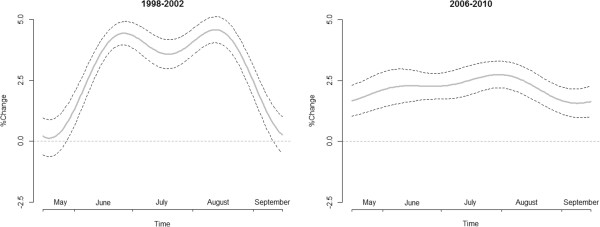
Overall meta-analytic time-varying effects (95% CI) of MAT on daily 65+ mortality (LAG 0–3) pre (1998–2002) and post (2006–2010) intervention.

Figure 4 shows the overall pre-post time-varying estimates. Two different patterns can be recognized in the two periods studied. Before the intervention, a bimodal trend can be observed, with one peak in June and the other in August. Conversely, after the intervention a more homogeneous effect throughout the season was observed; the two peaks are greatly reduced while in May and September greater effects on mortality were observed.

### Sensitivity Analysis

The sensitivity analysis confirmed the main analysis.

## Discussion

This is the first multicenter study investigating temporal variations of the heat/mortality relationship preceding and following the introduction of a national prevention plan of the effects of heat on health.

The main finding of our study was a significant decrease in the effect of high temperatures on mortality in the elderly (65+ years old) in the period following the implementation of the Italian national prevention plan, although some degree of heterogeneity between cities was observed. The decrease was observed only in correspondence with high temperatures, approximately above the 75^th^ percentile of city-specific temperature summer distribution, while no variations or increases in the effect on mortality were observed with lower temperatures.

Time-varying analysis results showed a reduction in the effect of temperatures in August and an increase in the months of May, June and September between the two periods. These changes over time may be explained by differences in the level of exposure, and changes in population susceptibility and adaptive capacity [2].

It should be considered that the introduction of city-specific HHWWS and prevention activities within the Italian heat Prevention Plan since 2004 might have improved the adaptive capacity of the population between the two study periods.

The national program is mainly focused on preventing the effects of extreme temperatures, in particular during heat waves, when level 2 and 3 warnings are issued and most prevention measures are activated, and media attention is high. However, when level 1 pre-warnings are issued, corresponding with milder temperatures, not all prevention activities are activated. Extreme temperatures are more frequent in the central months of the summer season, July and August, whereas level 1 pre-warnings are mainly issued at the beginning and the end of the summer. Furthermore it has to be considered that although HHWWS is operational in May and the public is alerted through the media, most prevention measures specifically targeted to susceptible subgroups are activated in June when the first hot episodes may have already occurred. This combination of factors might justify the different temporal trends observed in correspondence with mild and high temperatures.

Two other Italian studies have attempted to analyse temporal variations in the heat-mortality relationship [7,10], suggesting a decrease in the impact of high temperature on mortality in the years following 2003; however, in both cases the short study period might have affected the validity of the results. Similarly, at the international level, some studies have documented decreasing effects of heat on mortality that have been explained by an improvement in individual and infrastructure adaptation as a consequence of the introduction of HHWWS and prevention programs, an increase in population awareness, and the widespread use of home air conditioning [3-6,8,9,25,26].

The two other important factors that may contribute to the observed difference in the two periods are variations in the seasonality of the exposure and changes over time in the pool of susceptible subjects.

Although the period analysed is relatively short, results suggest that the temperature distribution has changed showing an increase in monthly temperatures in May, July and September since the activation of the program; this is coherent with national studies on climate change, which have suggested an increase in temperatures in Italy with an elongation of the summer season [27,28]. These variations might explain the increase in the effect of milder temperatures values which occur when a population is less prepared to cope with these conditions.

Concerning the second aspect, in more recent years an increase in the size of the susceptible population might have occurred as a consequence of the progressive aging of the population in the 16 cities. In Italy the percentage of persons over 65 increased from 18.0% in 1999 to 20.2% in 2009 [29]. This increase might explain the increment in the effect of high temperatures early in the summer period when the size of the susceptible population is greater.

One important point of strength of our analysis is the multicenter study design, and the associated statistical power of a large study. Although the 16 cities included in our study cover a wide range of climatic and socio-demographic conditions and the effect of heat is heterogeneous, we found a similar pattern of the heat-mortality relationship in most cities before and after the introduction of the program. Furthermore, the heterogeneity test was not significant in correspondence with the highest temperature values strengthening pooled/meta-analytic estimates. Sensitivity analyses performed to test the robustness of the pooled estimates gave largely unchanged results.

The use of DLNM ensured that temporal changes in the effect of temperature on mortality simultaneously accounts for the non-linearity of the studied relationship and the delayed effect of exposure [20]. This approach overcomes some of the main critical methodological aspects typical of the study of the heat/mortality relationship [21].

More frequently the temperature effect is modelled using a linear-threshold model where the threshold is derived from segmented regression. This method has been criticized, however, because it is sensitive to the selected threshold values [21]. Moreover, the use of a linear-threshold model signifies that the effect of heat on mortality is assumed constant for the whole temperature range above the threshold, simplifying the actual relationship, and the effect on mortality of temperatures below the threshold was not analysed. Temporal variations in air pollutant concentrations may have played a different role in explaining observed temporal trends of the temperature/mortality relationship in the two periods. However, in Italy air pollution levels have decreased in recent years thanks to EU air quality Directives [30], a reduction in the number of older and polluting cars, as well as a decline in daily use of transportation by car in response to the economic crisis. The reduction in the effects of air pollution is now evident in many countries [31,32]. This aspect was not addressed in this study. Airport station temperatures were used because they were the only standardised data source available at the national level. However, although there might be a possible misclassification in exposure due to the distance from the city centre, it should be constant throughout the time period considered, thus not limiting the comparability of the estimates in the two periods.

The time-varying analysis assumes linearity even if we know that the temperature-mortality relationship is different in the different ranges of temperature. While not ideal, it helps in forming a picture of the effect’s changes over the season in the two analysed periods. From this perspective, we think its application was useful.

Although the design used does not directly evaluate the effectiveness of heat prevention plan measures, pre-post analyses are widely used in other settings with similar limits for RCTs like in assessing policies to reduce air pollution [33], screening programs and routine immunization programs [34,35]. Every year at the national and local level considerable financial resources are devoted to heat-prevention plans’ implementation; however, as already mentioned, a formal evaluation has not yet been conducted and will be a challenging task. In this context, our study should be considered an indirect evaluation of the effectiveness of the Italian national heat prevention plan.

## Conclusions

In conclusion, our study provides evidence of a reduction in the effect of extreme summer temperatures on daily mortality in the 65+ year old population, attributable to variations in temperature distributions during summer, variations in the pattern of population susceptibility factors, and to the introduction of adaptation measures. In the study cities prevention programmes may have played a major role in mitigating the impact of high temperatures. However, an effect of mild temperature remains, indicating a relevant impact of temperature at the beginning and the end of the summer season. In order to further reduce the burden of deaths associated with heat, prevention plans should take into account variations in the heat-mortality relationship, and probably cover a wider period of program operations to include earlier and late summer periods when the effect of heat is becoming stronger.

## Abbreviations

MAT: maximum apparent temperature; RCT: Randomized Clinical Trials; HHWWS: Heat Health Watch Warning Systems; GPs: General Pratictioners; DLNM: Non-Linear Distributed Lag Model; GAM: Generalized Additive Model.

## Competing interests

The author declare that they no competing interests.

## Authors’ contributions

PS conceived, coordinated and designed the study, it and drafted the manuscript. ML designed the study and performed the statistical analysis and contributed to the draft of the manuscript. MDS helped draft the manuscript. FdD as expert on meteorological data contributed to the discussion of results and draft of the manuscript. AMB contributed to the discussion of results and helped draft the manuscript. DD and CM helped draft the manuscript. PM helped conceive the study and helped to draft the manuscript. All authors read and approved the final manuscript.

## Supplementary Material

Additional file 1 **Table 1.** City-specific percent change increase in daily 65+ yr mortality associated with 3°C increases in maximum apparent temperature (MAT) pre (1998–2002) and post intervention (2006–2010). **Figure 1.** Plots of city-specific effects (RR, 95%CI) on daily 65+ mortality associated with 3°C increases in MAT pre (1998–2002) and post (2006–2010) intervention. Click here for file
