# Supplementary material for: Changes in the effects of heat on mortality among the elderly from 1998–2010: results from a multicenter time series study in Italy
Source: Environ Health. 2012 Sep 3;11:58. doi: 10.1186/1476-069X-11-58 (PMC3506566; doi:10.1186/1476-069X-11-58)

Additional file 1 – web-only

Table 1_additional file. City-specific effects (% change, 95%CI) on daily 65+ mortality associated with 3°C increases in MAT pre (1998-2002) and post (2006-2010) intervention.

| **City** | **PRE 1998-2002** | | | | | | | | | | | | | | | | | | | |
| --- | --- | --- | --- | --- | --- | --- | --- | --- | --- | --- | --- | --- | --- | --- | --- | --- | --- | --- | --- | --- |
|  | **0°C to 3°C** | | | | **3°C to 6°C** | | | | **6°C to 9°C** | | | | **9°C to 12°C** | | | | **12°C to 15°C** | | | |
|  | **%** | **95% CI** | | | **%** | **95% CI** | | | **%** | **95% CI** | | | **%** | **95% CI** | | | **%** | **95% CI** | | |
| **Trieste** | 2.8 | -18.6 | ; | 29.6 | 5.7 | -15.6 | ; | 32.5 | 4 | -12.3 | ; | 23.2 | 7.9 | -11.6 | ; | 31.7 | 2.8 | -23.9 | ; | 38.7 |
| **Brescia** | 4.3 | -20.9 | ; | 37.7 | -7.2 | -32.4 | ; | 27.4 | 30.4 | -1.2 | ; | 72.1 | 16.1 | -7.7 | ; | 46.1 | -8.1 | -47.1 | ; | 59.6 |
| **Milano** | -4.1 | -18.8 | ; | 13.2 | 0.6 | -11.8 | ; | 14.7 | 17.2 | 4.3 | ; | 31.8 | 24.8 | -7.1 | ; | 67.8 | 26.7 | -13 | ; | 84.6 |
| **Verona** | 8.3 | -23.2 | ; | 52.7 | 3.7 | -28.8 | ; | 50.9 | -10.9 | -36.3 | ; | 24.7 | 35 | -2.9 | ; | 87.6 | 68.2 | -13 | ; | 225.3 |
| **Venezia** | 8.6 | -14.1 | ; | 37.2 | -10 | -29.1 | ; | 14.2 | 25.1 | 4.5 | ; | 49.8 | 15.2 | -0.2 | ; | 33 | -4.1 | -28.4 | ; | 28.4 |
| **Torino** | 1.6 | -12.6 | ; | 18 | 3.9 | -15.2 | ; | 27.4 | 15.8 | -4.6 | ; | 40.7 | 10.9 | -10.4 | ; | 37.3 | 6.1 | -29.4 | ; | 59.4 |
| **Bologna** | -7.9 | -30.5 | ; | 22 | 20.5 | -14 | ; | 68.8 | 18.1 | -11.2 | ; | 57 | 39.6 | -15.1 | ; | 129.6 | 45.4 | -24.4 | ; | 179.8 |
| **Genova** | 3.7 | -8.3 | ; | 17.3 | 23.7 | 12.1 | ; | 36.4 | 14.1 | 6.5 | ; | 22.2 | -7.1 | -22.2 | ; | 11.1 | -12.5 | -30 | ; | 9.5 |
| **Firenze** | -5.3 | -27.2 | ; | 23.1 | 25.3 | -1.1 | ; | 58.8 | 4.3 | -14.1 | ; | 26.7 | 5.5 | -33 | ; | 66.1 | 6.1 | -42 | ; | 94.1 |
| **Viterbo** | -22 | -54.5 | ; | 33.5 | 33.4 | -32.8 | ; | 164.9 | 3.7 | -52.3 | ; | 125.2 | -3.8 | -63 | ; | 149.8 | 1.7 | -86.4 | ; | 659.8 |
| **Civitavecchia** | -8.6 | -54.8 | ; | 85 | 14.7 | -56 | ; | 198.6 | 23.8 | -58.8 | ; | 271.9 | -12.4 | -62.8 | ; | 106.1 | -30.2 | -92 | ; | 512.8 |
| **Roma** | 15.5 | 6.4 | ; | 25.3 | -7.1 | -16 | ; | 2.7 | 23.6 | 11.4 | ; | 37.2 | 15.9 | 1.5 | ; | 32.2 | 4 | -20.2 | ; | 35.4 |
| **Campobasso** | 33.2 | -29.4 | ; | 151.6 | -28.4 | -63.6 | ; | 40.9 | 70.3 | 5.3 | ; | 175.4 | -1.9 | -42 | ; | 65.9 | -64.6 | -89.7 | ; | 21.5 |
| **Latina** | -19 | -50.9 | ; | 33.6 | 58 | -10.5 | ; | 179 | -33.7 | -61.7 | ; | 15.1 | 56.2 | -28.4 | ; | 240.8 | 154.6 | -35.5 | ; | 905.1 |
| **Bari** | 21.1 | -14.4 | ; | 71.3 | 17.3 | -13.7 | ; | 59.4 | 39.1 | 5.4 | ; | 83.7 | 92.8 | 3.4 | ; | 259.6 | 115.8 | -7.9 | ; | 405.3 |
| **Palermo** | -7.5 | -26.3 | ; | 16 | 24.8 | -0.7 | ; | 56.8 | 6.4 | -13.3 | ; | 30.7 | 24.4 | -3.3 | ; | 60 | 33.5 | -10.9 | ; | 100.1 |

(to be continued)

| **City** | **POST 2006-2010** | | | | | | | | | | | | | | | | | | | |
| --- | --- | --- | --- | --- | --- | --- | --- | --- | --- | --- | --- | --- | --- | --- | --- | --- | --- | --- | --- | --- |
|  | **0°C to 3°C** | | | | **3°C to 6°C** | | | | **6°C to 9°C** | | | | **9°C to 12°C** | | | | **12°C to 15°C** | | | |
|  | **%** | **95% CI** | | | **%** | **95% CI** | | | **%** | **95% CI** | | | **%** | **95% CI** | | | **%** | **95% CI** | | |
| **Trieste** | 2.8 | -18.6 | ; | 29.6 | 5.7 | -15.6 | ; | 32.5 | 4 | -12.3 | ; | 23.2 | 7.9 | -11.6 | ; | 31.7 | 2.8 | -23.9 | ; | 38.7 |
| **Brescia** | 4.3 | -20.9 | ; | 37.7 | -7.2 | -32.4 | ; | 27.4 | 30.4 | -1.2 | ; | 72.1 | 16.1 | -7.7 | ; | 46.1 | -8.1 | -47.1 | ; | 59.6 |
| **Milano** | -4.1 | -18.8 | ; | 13.2 | 0.6 | -11.8 | ; | 14.7 | 17.2 | 4.3 | ; | 31.8 | 24.8 | -7.1 | ; | 67.8 | 26.7 | -13 | ; | 84.6 |
| **Verona** | 8.3 | -23.2 | ; | 52.7 | 3.7 | -28.8 | ; | 50.9 | -10.9 | -36.3 | ; | 24.7 | 35 | -2.9 | ; | 87.6 | 68.2 | -13 | ; | 225.3 |
| **Venezia** | 8.6 | -14.1 | ; | 37.2 | -10 | -29.1 | ; | 14.2 | 25.1 | 4.5 | ; | 49.8 | 15.2 | -0.2 | ; | 33 | -4.1 | -28.4 | ; | 28.4 |
| **Torino** | 1.6 | -12.6 | ; | 18 | 3.9 | -15.2 | ; | 27.4 | 15.8 | -4.6 | ; | 40.7 | 10.9 | -10.4 | ; | 37.3 | 6.1 | -29.4 | ; | 59.4 |
| **Bologna** | -7.9 | -30.5 | ; | 22 | 20.5 | -14 | ; | 68.8 | 18.1 | -11.2 | ; | 57 | 39.6 | -15.1 | ; | 129.6 | 45.4 | -24.4 | ; | 179.8 |
| **Genova** | 3.7 | -8.3 | ; | 17.3 | 23.7 | 12.1 | ; | 36.4 | 14.1 | 6.5 | ; | 22.2 | -7.1 | -22.2 | ; | 11.1 | -12.5 | -30 | ; | 9.5 |
| **Firenze** | -5.3 | -27.2 | ; | 23.1 | 25.3 | -1.1 | ; | 58.8 | 4.3 | -14.1 | ; | 26.7 | 5.5 | -33 | ; | 66.1 | 6.1 | -42 | ; | 94.1 |
| **Viterbo** | -22 | -54.5 | ; | 33.5 | 33.4 | -32.8 | ; | 164.9 | 3.7 | -52.3 | ; | 125.2 | -3.8 | -63 | ; | 149.8 | 1.7 | -86.4 | ; | 659.8 |
| **Civitavecchia** | -8.6 | -54.8 | ; | 85 | 14.7 | -56 | ; | 198.6 | 23.8 | -58.8 | ; | 271.9 | -12.4 | -62.8 | ; | 106.1 | -30.2 | -92 | ; | 512.8 |
| **Roma** | 15.5 | 6.4 | ; | 25.3 | -7.1 | -16 | ; | 2.7 | 23.6 | 11.4 | ; | 37.2 | 15.9 | 1.5 | ; | 32.2 | 4 | -20.2 | ; | 35.4 |
| **Campobasso** | 33.2 | -29.4 | ; | 151.6 | -28.4 | -63.6 | ; | 40.9 | 70.3 | 5.3 | ; | 175.4 | -1.9 | -42 | ; | 65.9 | -64.6 | -89.7 | ; | 21.5 |
| **Latina** | -19 | -50.9 | ; | 33.6 | 58 | -10.5 | ; | 179 | -33.7 | -61.7 | ; | 15.1 | 56.2 | -28.4 | ; | 240.8 | 154.6 | -35.5 | ; | 905.1 |
| **Bari** | 21.1 | -14.4 | ; | 71.3 | 17.3 | -13.7 | ; | 59.4 | 39.1 | 5.4 | ; | 83.7 | 92.8 | 3.4 | ; | 259.6 | 115.8 | -7.9 | ; | 405.3 |
| **Palermo** | -7.5 | -26.3 | ; | 16 | 24.8 | -0.7 | ; | 56.8 | 6.4 | -13.3 | ; | 30.7 | 24.4 | -3.3 | ; | 60 | 33.5 | -10.9 | ; | 100.1 |

Figure 1-_additional file_. Plots of city-specific effects (RR, 95%CI) on daily 65+ mortality associated with 3°C increases in MAT pre (1998-2002) and post (2006-2010) intervention.


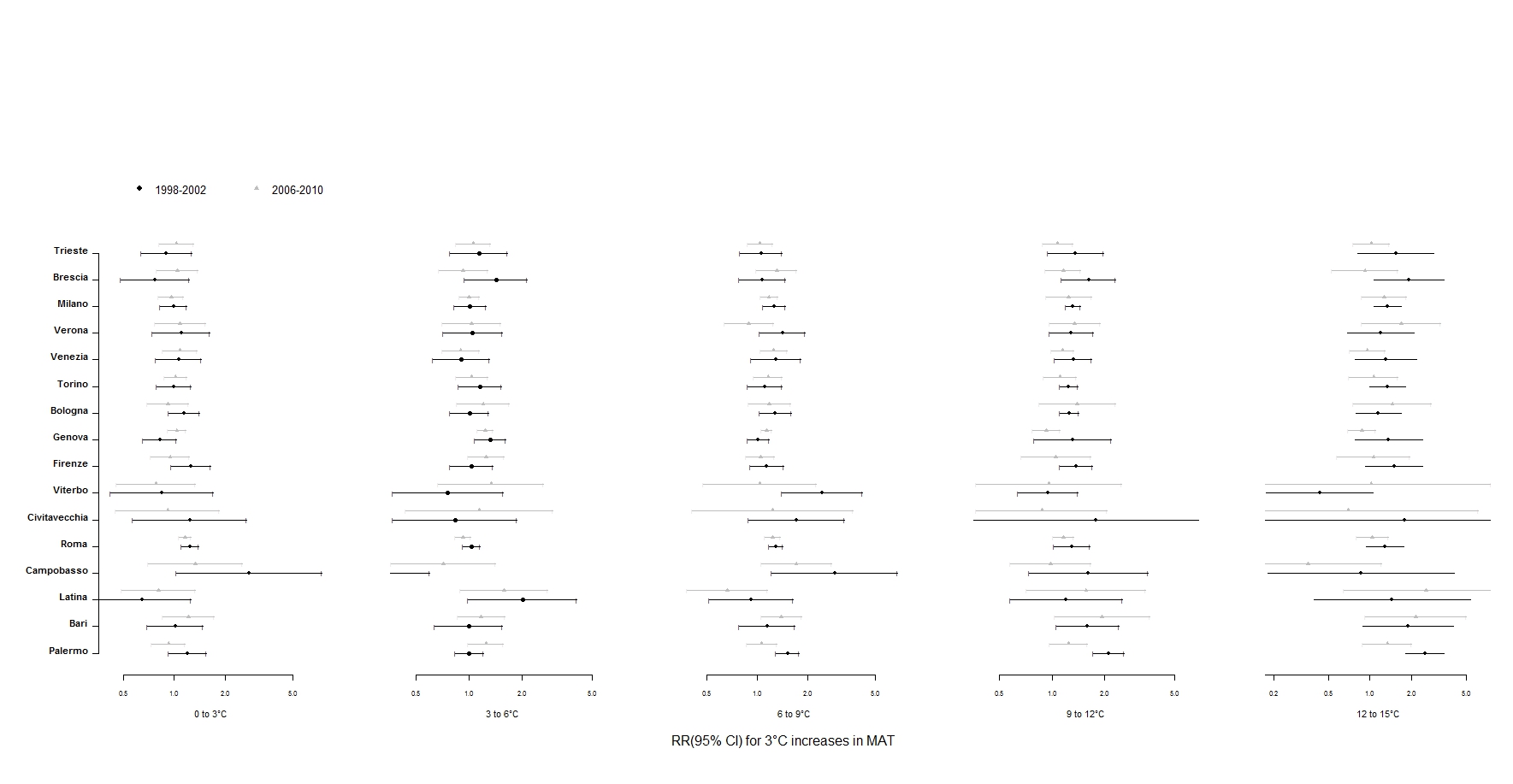

Supplement: Additional file 1 — Table 1. City-specific percent change increase in daily 65+ yr mortality associated with 3°C increases in maximum apparent temperature (MAT) pre (1998–2002) and post intervention (2006–2010). Figure 1. Plots of city-specific effects (RR, 95%CI) on daily 65+ mortality associated with 3°C increases in MAT pre (1998–2002) and post (2006–2010) intervention. [file 1476-069X-11-58-S1.doc]
